# Studying patterns and predictors of HIV viral suppression using A Big Data approach: a research protocol

**DOI:** 10.1186/s12879-022-07047-5

**Published:** 2022-02-04

**Authors:** Jiajia Zhang, Bankole Olatosi, Xueying Yang, Sharon Weissman, Zhenlong Li, Jianjun Hu, Xiaoming Li

**Affiliations:** 1 Department of Epidemiology and Biostatistics, Arnold School of Public Health, University of South Carolina, Columbia, SC 29208 USA; 2 South Carolina SmartState Center for Healthcare Quality, Arnold School of Public Health, University of South Carolina, Columbia, SC 29208 USA; 3 Big Data Health Science Center (BDHSC), University of South Carolina, Columbia, SC 29208 USA; 4 Department of Health Services Policy and Management, Arnold School of Public Health, University of South Carolina, Columbia, SC 29208 USA; 5 Department of Health Promotion, Education and Behavior, Arnold School of Public Health, University of South Carolina, Columbia, SC 29208 USA; 6 Department of Internal Medicine, School of Medicine, University of South Carolina, Columbia, SC 29208 USA; 7 Geoinformation and Big Data Research Laboratory, University of South Carolina, Columbia, SC 29208 USA; 8 Department of Computer Science and Engineering, University of South Carolina, Columbia, SC 29208 USA

**Keywords:** HIV/AIDS, Viral suppression, Viral rebound, Pattern analysis, Data analytics

## Abstract

**Background:**

Given the importance of viral suppression in ending the HIV epidemic in the US and elsewhere, an optimal predictive model of viral status can help clinicians identify those at risk of poor viral control and inform clinical improvements in HIV treatment and care. With an increasing availability of electronic health record (EHR) data and social environmental information, there is a unique opportunity to improve our understanding of the dynamic pattern of viral suppression. Using a statewide cohort of people living with HIV (PLWH) in South Carolina (SC), the overall goal of the proposed research is to examine the dynamic patterns of viral suppression, develop optimal predictive models of various viral suppression indicators, and translate the models to a beta version of service-ready tools for clinical decision support.

**Methods:**

The PLWH cohort will be identified through the SC Enhanced HIV/AIDS Reporting System (eHARS). The SC Office of Revenue and Fiscal Affairs (RFA) will extract longitudinal EHR clinical data of all PLWH in SC from multiple health systems, obtain data from other state agencies, and link the patient-level data with county-level data from multiple publicly available data sources. Using the deidentified data, the proposed study will consist of three operational phases: Phase 1: “Pattern Analysis” to identify the longitudinal dynamics of viral suppression using multiple viral load indicators; Phase 2: “Model Development” to determine the critical predictors of multiple viral load indicators through artificial intelligence (AI)-based modeling accounting for multilevel factors; and Phase 3: “Translational Research” to develop a multifactorial clinical decision system based on a risk prediction model to assist with the identification of the risk of viral failure or viral rebound when patients present at clinical visits.

**Discussion:**

With both extensive data integration and data analytics, the proposed research will: (1) improve the understanding of the complex inter-related effects of longitudinal trajectories of HIV viral suppressions and HIV treatment history while taking into consideration multilevel factors; and (2) develop empirical public health approaches to achieve ending the HIV epidemic through translating the risk prediction model to a multifactorial decision system that enables the feasibility of AI-assisted clinical decisions.

## Background

Viral suppression is the final stage of the HIV treatment cascade, which serves as the framework for UNAIDS’ 90–90–90 goals [1]. Sustained (or durable) viral suppression permits the restoration of immune function, reduces onward secondary transmission, and indicates long-term treatment success and mortality reduction [2]. In the US, ~ 57% of all people living with HIV (PLWH) were virally suppressed according to the national surveillance data from the Centers for Disease Control and Prevention (CDC) [3], and in South Carolina (SC), 62% of PLWH were virally suppressed [4]. “Ending the HIV Epidemic (EtHE): A Plan for America” [5] federal campaign, launched in February 2019 aims to reduce the number of new HIV infections in the US by 75% and 90% by 2025 and 2030, respectively. The *EtHE* campaign focuses on 48 US counties that contribute to > 50% of new HIV cases and 7 states with a high rural HIV burden, including SC. Sustained viral suppression is one of four strategic areas of the *EtHE.* With a prolonged life expectancy of PLWH, routine monitoring of viral load (VL) status becomes more important over their life course, with the longitudinal VL information collected over time potentially adding to the predictability of subsequent virologic failure (VF) or mortality. Over the past few years, a small but increasing number of longitudinal studies have explored the dynamics of VL patterns using sustained viral suppression, viral rebound, viral blips, or low-level viremia (LLV) [6–8]. These different VL measures are interrelated, affect each other, and also predict, to some extent, virologic failure [9]. Studies examining the association between persistent LLV and VF or viral rebound are conflicting [10–12]. Other studies report a correlation between LLV and the risk of viral rebound [13–15]. Furthermore, the threshold of LLV at which it would be predictive of VF varies. Some studies suggest a threshold of > 200 copies/ml as being associated with VF; yet other studies suggest a higher threshold (i.e., VL > 400 copies/ml) [16, 17].

The virological outcomes of PLWH could be affected by multiple factors from individual-level (e.g., socio-demographics, clinical characteristics, HIV care-seeking behaviors) to county-level social and environmental factors (e.g., economic environment). Socio-demographics have been frequently reported to be associated with viral suppression. According to the US CDC’s HIV Prevention Progress Report 2019, viral suppression remains lowest among persons ≤ 34 years, Blacks/African Americans, persons who inject drugs, and heterosexuals [18, 19]. The clinical indicators of HIV diagnosis (e.g., pre-antiretroviral therapy [ART] CD4 counts, pre-ART VL level) are important in determining subsequent virologic success or failure after initiation of ART [16, 19–21]. Individuals who are more immunocompromised (e.g., low baseline CD4 counts, or opportunistic illnesses) at HIV diagnosis are more likely to develop VF [16, 19–21]. Treatment history, including earlier ART initiation [19], no prior ART use before treatment [20], prior use of mono- or dual- antiretrovirals [22], longer duration of therapy [20], and boosted protease inhibitor (PI)-based regimen [20], also impacts success or failure of sustained viral suppression [16, 21]. Apart from individual-level factors, considerable interest remains in understanding how social and structural determinants of health affect the HIV treatment continuum, including viral suppression. For instance, the structural determinants and socioeconomic conditions of the neighborhoods or communities (e.g., county) where individual lives will profoundly impact the outcomes of the HIV continuum of care [23–25]. A more comprehensive prediction model for virologic outcomes based on the dynamic patterns of VL, individual demographics, HIV care-seeking behavior, and social and environmental factors, could inform us on “when” and “how” to help individuals with poor viral control to achieve and sustain viral suppression.

Some critical gaps exist in the current efforts to understand the dynamics of viral suppression and the development of an optimal predictive model of viral suppression. First, most studies have focused on limited indicators of viral suppression (e.g., a single time point measure) and have failed to provide a complete picture of the dynamic process of viral suppression. Second, most studies investigating the transformations among virologic outcomes have only explored the monotonous transformation between two virologic outcomes (e.g., from viral suppression/failure to viral failure/suppression) rather than the comprehensive virologic history and dynamic viral patterns [26]. Third, most studies have examined viral suppression within a limited time window ranging from 6 to 48 months [26, 27] and were unable to provide a time-sensitive assessment of the virial suppression process. Fourth, scopes of data in most existing studies are insufficient to fully describe the viral patterns due to limited data sources (e.g., use only medical records or epidemiological surveys). The structural and socioenvironmental factors were not always taken into consideration because of either the unavailability of such data in medical records or the lack of advanced analytic tools that can model such complex data. Fifth, previous studies lacked advanced analytics to predict the VF (or other viral outcomes) using the comprehensive and longitudinal data. Most extant literature counted the presence of virologic outcomes within a limited timeframe and explored their correlates using traditional analytic approaches such as generalized estimate equation [28] and cox regression [7, 8, 29]. Most of these approaches are time insensitive and cannot make dynamic predictions based on the large representative features. Finally, most of the existing research does not go beyond the modeling phase to translate research findings into service-ready clinical tools for improved viral suppression or better viral control.

## Methods/design

### Objective

Using a data science approach, this study aims to examine the longitudinal dynamic pattern of viral suppression, develop optimal predictive models of various viral suppression indicators, and translate the models to service-ready tools for clinical support and decision-making. Our main research objectives are threefold. The first objective is to identify the longitudinal dynamics of viral suppression among PLWH in SC using multiple indicators, including, but not limited to, time to initial suppression (from diagnosis or ART uptake), sustained suppression (e.g., virally suppressed for > 40 months), viral rebound (both time to rebound and level of viral rebound), viral blips, and other relevant VL measures (e.g., LLV). The second objective is to determine the critical predictors of multiple VL indicators through artificial intelligence (AI)-based modeling accounting for factors at the individual level (e.g., patient demographics, treatment regimen, and health care service utilization), structural level (e.g., geographic region, availability of treatment facility, and specialty), and socioenvironmental level (e.g., socioeconomic level). Finally, the research will develop a multifactorial decision system based on a risk prediction model to assist with the identification of the risk of VF or viral rebound when patients present at clinical visits.

### Conceptual framework

**Fig. 1:**
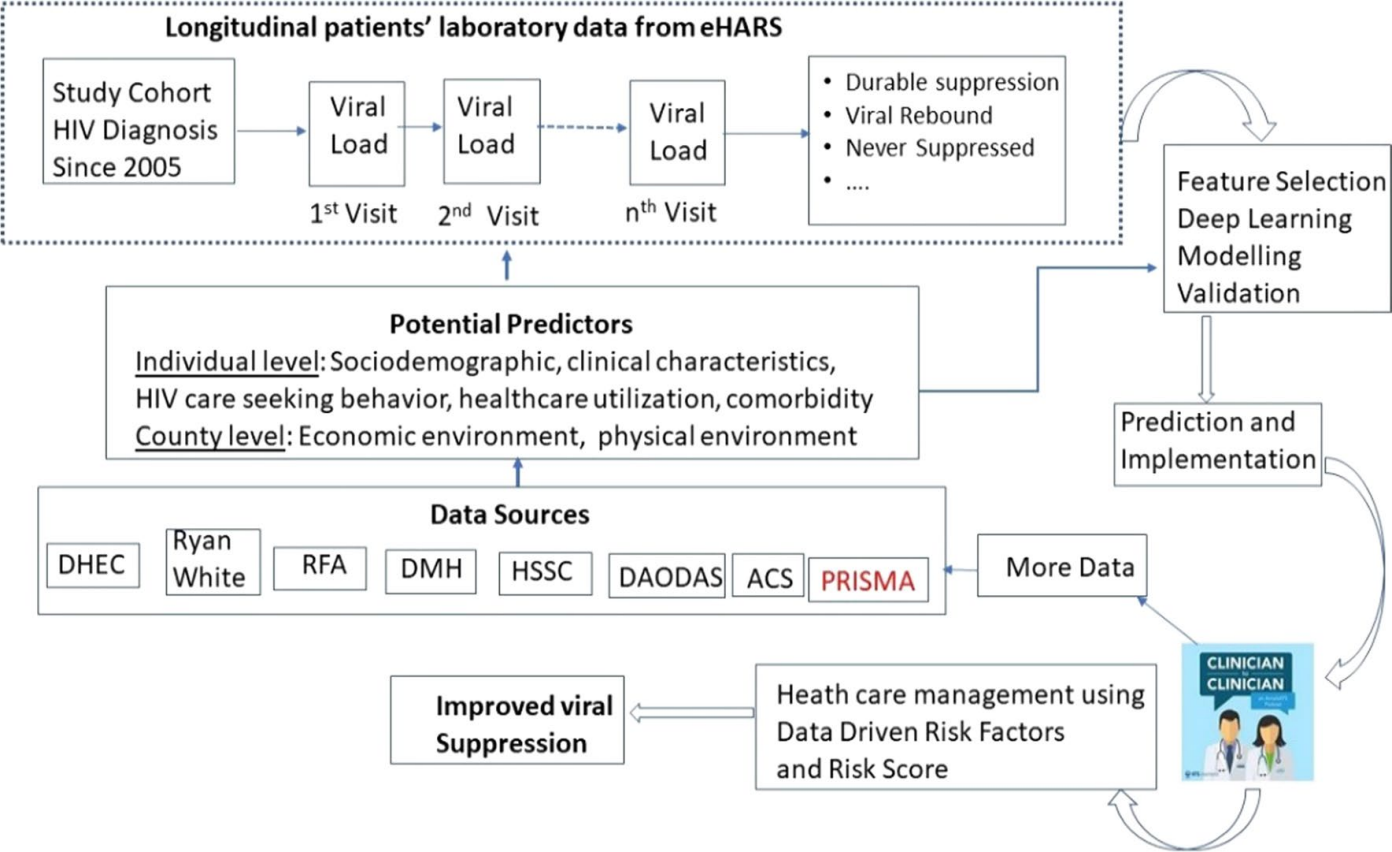
Conceptual model of the proposed research

Since 2017, we have been utilizing a data science approach to examine treatment gaps among PLWH in SC [30, 32]. The ongoing research extracted longitudinal electronic health records data of all PLWH in SC from multiple state agencies and health systems. We linked the individual-level data with social environmental data (e.g., social economics, number of health care professionals, hospitals, and health care facilities) from multiple publicly available data sources. The integrated database enabled us to successfully “track” 11,470 patients who were diagnosed with HIV in 2005–2016 in SC and identify the gaps (e.g., missed opportunities) in HIV treatment linkage and retention [33]. Guided by a conceptual framework (Fig. 1), the proposed study will (1) continue to “follow” our cohort for another five years (and also expand the cohort by adding PLWH diagnosed between 2016 and 2020); (2) expand our database to include additional treatment and laboratory data from the newly established Prisma Health system that serves about 1/2 of the state’s population; (3) expand our database to include additional data on alcohol and other drug use, and (4) employ AI-based modeling to understand the dynamic VL patterns (e.g. VF/suppression/rebound/blip and LLV) and their predictors.

### Population and setting

The proposed study will be conducted in SC, which is one of the states bearing the highest burden of HIV in the nation. SC has consistently ranked among the top ten US states in the number of annual HIV/AIDS cases during the past several years [34, 35]. As of December 31, 2019, there were 20,334 SC residents living with HIV, and this number has increased by 30% since 2008. With an incidence rate of 15.5 per 100,000, SC has the 8th highest incidence rate of HIV infection nationally [36]. The proposed study is responding to the aims of the *EtHE* campaign [5] by addressing the NIH HIV/AIDS priority topic areas for HIV treatment outcomes. The PLWH population in SC is diverse in terms of gender (72% male), and race/ethnicity (68% African American, 25% White, 5% Hispanic/Latino) [36]. This distribution represents the general characteristics of HIV populations in most Southern states. Thus, ensuring that the population-based results will have strong generalizability and can provide timely evidence/guidance on HIV treatment strategies among PLWH in SC and other places where relevant data are available. The study population of the proposed research includes people who were diagnosed with HIV in SC between 2005 and 2020. Only people aged ≥ 18 years in the year of diagnosis are included in the analyses. We chose 2005 because this was the year after the state law for mandatory reporting of all CD4 and VL tests to e-HARS began. At least fifteen years (2005–2020) of HIV utilization data will be available for this study. Additional utilization data will be collected and included during annual data updates up till 2025.

### Data sources and data acquisition and management

The increasing availability of electronic data, including electronic medical records, administrative databases, and public county-level data, has created a unique opportunity to expand our ability to measure HIV-related health and clinical outcomes. The proposed study will integrate data from both individual-level and county-level. Using the integrated data, we can examine patients’ risk factors at the individual and county levels longitudinally and generate new knowledge of HIV viral suppression. Data sources are described in greater detail below.

### Individual-level data sources

The individual level data are integrated from eight state agencies/systems, including: (1) SC Enhanced HIV/AIDS Reporting System (eHARS) [37, 38]; (2) Ryan White HIV/AIDS Program Data Report (RDR); (3) SC Revenue and Fiscal Affairs Office (SC RFA) integrated data warehouse [38]; (4) Health Sciences South Carolina (HSSC); (5) SC Department of Mental Health (SCDMH); (6) SC Department of Corrections; (7) SC Department of Alcohol and Other Drug Abuse Services (DAODAS); and (8) Prisma Health System. Detailed descriptions of the data sources #1-#4 can be found elsewhere [33]. Below, we list the description of the additional new data sources #5–#8 for the study.

#### SC Department of Mental Health (SCDMH)

SCDMH comprises of 17 community-based outpatient mental health centers, with 60 satellite offices. The SCDMH’s community mental health system’s geographical areas include 17 centers where all mental health services are provided. More than 20 SCDMH sites provide specialized clinical care, including forensic and sexually violent predator treatment programs. SCDMH staff regularly provide clinical services in > 140 non-SCDMH facilities, including jails and SC Department of Social Services sites. Twenty-two community hospital emergency departments (EDs) utilize technology directly linking ED patients to a SCDMH psychiatrist for face-to-face behavior health consultation via video.

#### SC Department of Corrections

The SC Department of Corrections was established in 1960 and includes historical data on inmates’ criminal history which is also housed within the SC RFA integrated data warehouse. This study will include all SC department of corrections data from 2000 to 2020 relevant to PLWH.

#### SC Department of Alcohol and Other Drug Abuse Services (DAODAS)

DAODAS is the SC government agency responsible for providing services to prevent or reduce consequences of substance use and addictions. DAODAS contracts with 32 local alcohol and drug abuse authorities to ensure that prevention and treatment services are available across all 46 counties in SC. DAODAS data include client data on admissions/intake assessment, history of use, transfer, services provided, and discharge information related to alcohol and substance use among individual patients.

#### Prisma Health System

In 2018, two major SC health care systems (Palmetto Health, Greenville Health) merged to form Prisma Health, which is now the clinical partner of University of South Carolina’s (UofSC) two medical schools. As SC’s largest, not-for-profit organization, Prisma Health serves more than 1.2 million patients annually and treats about one-third of all Medicaid patients statewide. It is estimated that 1 of every 2 SC residents’ lives within a 15-min drive of a Prisma/UofSC Medical Group facility, which includes 13 hospitals. The Prisma health system employs an estimated 32,000 health care providers and workers, making it the largest private employer in SC. Because of this unparalleled reach, Prisma Heath is uniquely positioned to drive improvements in clinical care across SC.

### County-level data sources

#### American Community Survey (ACS)

The ACS is a nationwide survey from a sample of the population in the US and Puerto Rico [40]. The ACS collects information such as age, race, income, education, and other socioeconomic/demographic data. All ACS data are survey estimates, and each estimate has a margin of error published by the US Census Bureau. ACS estimates are period estimates that describe the average of characteristics of the population and housing over a period of data collection.

#### Area health resources file (AHRF)

AHRF is a public county-level dataset from HRSA that contains files in eight domains namely: Health Care Professions, Health Facilities, Population Characteristics, Economics, Health Professions Training, Hospital Utilization, Hospital Expenditures, and Environment. AHRF was designed to be used by policymakers, researchers, and others interested in the nation’s health care delivery systems and factors that may impact health status and health care in the US.

#### Behavioral risk factor surveillance system (BRFSS)

BRFSS is a CDC-funded state-based system of surveys that collects information on health-risk behaviors, preventive health practices, and health care access. It is a key source of tobacco use, alcohol consumption, and cancer screening data at the county level.

#### County health rankings and roadmaps program

The County Health Rankings & Roadmaps program is a collaboration between the Robert Wood Johnson Foundation and the University of Wisconsin Population Health Institute. It measures vital health factors, including high school graduation rates; obesity; smoking; unemployment; access to healthy foods, air and water quality; and income inequality at the county level.

### Data acquisition and management

Following a similar protocol in our ongoing Big Data analytic research in SC, we will establish a legal contract (which is required for each new study or new analysis) with SC RFA that will serve as the honest broker for the linkage of all identifiable data. The SC RFA will remove all the identifiable information from the linked data before releasing it to the research team. The detailed data acquisition and linkage process were described previously elsewhere [33]. In the proposed study, we will update the database in our ongoing project by including (1) additional adult PLWH who were diagnosed between 2016 and 2020; (2) additional treatment and laboratory data from Prisma Health; and (3) additional alcohol and other substance use data from DAODAS for the entire study cohort. Specifically, all participating SC agencies will submit their EHR data of the PLWH cohort to SC RFA. The SC RFA will link patient records from all sources and generate a linked dataset, which will include longitudinal observations of hospital visits, medication, claims data, mental health visits, and other relevant data for the study cohort [41]. SC RFA will also link the aggregate county-level indicators with the patient-level data by the county code. In compliance with HIPAA regulations, SC RFA will create unique, non-identifiable client-level identifiers for this data linkage. The SC RFA de-identified system-generated number ensures confidentiality but allows the study to conduct data mining at both the individual and aggregated data levels. For data security, only the final, deidentified dataset will be released to the research team for analysis. With the deidentified data, the research team is responsible for carrying out further data management, data cleaning, and development of a data dictionary, following similar protocols we have established in our ongoing NIH funded studies for data management, storage, and security [33].

### Key study variables

#### Individual-level variables

The individual-level variables include sociodemographic characteristics (e.g., age at HIV diagnosis, gender, race, ethnicity, rural/urban area of residence, and poverty indicators such as patients’ eligibility status for Ryan White (RW) funding; HIV infection history (e.g., HIV diagnosis date; AIDS diagnosis date; source of report; transmission modes); longitudinal measures of CD4 counts (e.g., initial CD4 counts, nadir CD4 counts, recent CD4 counts, percentage of low CD4 counts); [42, 43]) HIV treatment cascade outcomes (e.g., linkage to care, retention in care) [44]; Longitudinal ART indicators (e.g., duration of ART, specific ART regimens, drug classes [NRTI-based, NNRTI-based, PI-based, or multi-class regimen with 3 or more classes of ART], regimens switch); Medical Conditions**.** A variety of clinical medical conditions will be measured using the ICD 9 or ICD10 diagnosis codes contained in the EHR data from SC RFA, Prisma and HSSC, such as comorbidity [45, 46], mental health disorders, and substance use and abuse.

#### County-level social-environmental variables

##### Neighborhood social environment scale

The following components of the scale will be used: (1) commercial stores, including pharmacies, beauty salons/barber shops, laundry/dry cleaner, supermarket; (2) population SES (per capita income, white-collar employees, crowding); (3) environment or housing (population of Census tract, area of Census tract, renters, single-family dwellings); and )4) average household size and % of female-headed households [47, 48]. Economic Environment Variables, including poverty rate, health coverage, median income, median home value, and social deprivation index (percent with less than 12 years of education, percent single parent household, percent living in rented housing unit, percent living in overcrowded housing unit, percent of households without a car, percent of non-employed adults under 65 years of age). Health Care Facility Data. Health facility data for the whole of SC will be obtained from SC DHEC, which contains health-side and environment-side information of health care facilities licensed by SC DHEC. Physical Environment Variables, including park information (county park, local park, national park or forest, regional park, and state park or forest), recreational area (amusement park, beach, golf course, and park and recreation area), etc. In addition, County-level Residential Data at three time points (HIV diagnosis, AIDS diagnosis, and current address) are available to identify mobility change and environmental change.

### Data analytics

#### Phase 1: pattern analysis

*Definitions of various VL Indicators.* Following the “US Guidelines for the Use of Antiretroviral Agents in Adults and Adolescents with HIV”, [49] the proposed measures of VL indicators (both time-point and longitudinal measures) and their operational or clinical definitions are displayed in Table 1, such as viral suppression, virologic failure, viral rebound, viral blip, and LLV. For example, sustained viral suppression is defined generally as a viral load persistently (e.g., ≥ 40 months) below the level of detection depending on the assay used (e.g., 200 copies/ml); viral rebound is defined as confirmed HIV RNA level ≥ 200 copies/ml after initial viral suppression.

**Table 1:** Multiple viral load (VL) indicators and their definitions

Time-point measure	Longitudinal measure
Viral suppression: a confirmed HIV RNA level below 200 copies/ml •Initial VL at HIV diagnosis •The current/most recent VL Viral rebound: confirmed HIV RNA level ≥ 200 copies/ml after viral suppression •Most recent viral rebound Viral failure: the inability to achieve or maintain suppression of viral replication to an HIV RNA level < 200 copies/ml Viral blip: After viral suppression, an isolated detectable HIV RNA level (≥200 copies/ml) that is followed by a return to viral suppression Low-level viremia: Confirmed detectable HIV RNA level <1000 copies/ml (at least two consecutive VL measures above 1000 copies/ml)	Aggregate feature: •Nadir VL •Peak VL •Number of viral rebounds •Size of the viral rebound (none, 500–1000, 1000–10,000 and > 10,000 copies/ml) Longitudinal feature: •Time to initial viral suppression •Time since the most recent viral rebound •Sustained viral suppression: patients with VL< 200 copies/ml in every VL measurement throughout the study period •Proportion of time spent with viral suppression (< 200 copies/ml) •Level of viral rebound (low level: at least 2 VL values were 500–5000 copies/ml; high-level: at least 2 VL values were >500 copies/ml) •Intermittent LLV: VL of 200–1000 copies/ml on < 25% of measurements •Persistent LLV: VL of 200–1000 copies/ml on ≥ 25% of measurements

To achieve the goals for phase 1, deep learning models (e.g., multilayer perceptron networks, convolutional neural network [CNN], and long short-term memory [LSTM] recurrent neural networks [RNN]), which have unique advantages in their modeling flexibility, will be employed to identify the common VL patterns based on our proposed predictors. Five virologic outcomes (viral suppression, viral rebound, viral blip, LLV, and VF), measured with dynamic temporal features will be used for unsupervised deep learning to identify the common patterns among PLWH in SC. The CNN, a deep learning model, is particularly suited to learning local patterns in raw input features, such as the sociodemographic characteristics and ART regimen. The modelling procedure includes data preprocessing and feature extraction, model training, model evaluation, and pattern interpretation. In the feature selection step, appropriately incorporating different longitudinal observations of VL measures into the pattern analysis is critical. We will use a prediction approach that will be jointly modelled with primary outcomes. The data will then be split into training and testing sets based on the ratio of 8:2. VL patterns will be abstracted from the convolution kernels of the CNN model and represented by the input patches that activate the feature maps most, which are the responses of the convolution kernels to the inputs. To see whether there is a significant difference in the performance, we will perform a paired t-test with the level of significance α = 0.05.

#### Phase 2: prediction modeling

The process of model development for phase 2 includes: (1) preparing the benchmark data and the process of task generation along with evaluation metrics; (2) developing neural baseline models for the benchmark tasks, the experimental setup and model selection, and (3) multitask learning.

#### Benchmark tasks

Considering the multiple VL indicators, we anticipate several tasks for prediction. Task 1 involves prediction of VL status among PLWH in SC. Viral load status, including viral suppression, LLV, rebound, blip and VF, is the primary outcome of interest. All viral load status will be defined as a binary outcome, and a supervised RNN will be employed to construct the classification model of viral load status. Task 2 involves the duration of suppression. Tasks 3 and 4 involve the time to suppression (failure) or rebound within 3 to 9 months since PLWH in SC will have their regular check-ups every 6 months. We will summarize the duration in suppression or time to suppression (failure) or rebound based on 5 quantiles, including minimum, 25th percentile, 50th percentile, 75th percentile, and maximum. According to these timeframes, we will design the time windows for modelling. This converts time into an ordinal multiclass classification problem. The Cohen’s linear weighted kappa will be used to measure correlation between ordered items.

#### Long short-term memory (LSTM) neural network

**Fig. 2:**
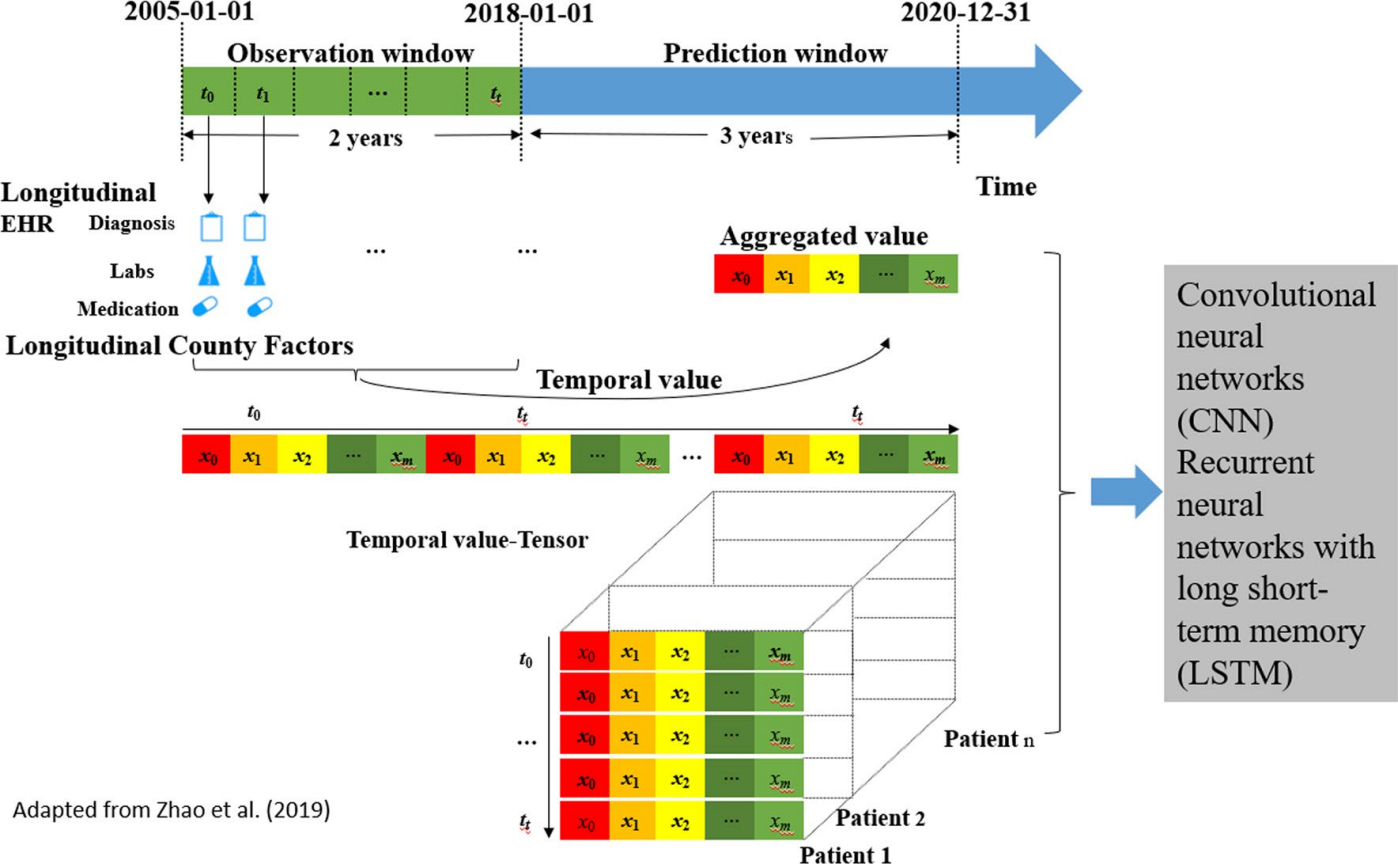
Risk prediction model

A LSTM neural network is a type of recurrent neural networks (RNNs) designed to capture long dependencies in sequential data. LSTM can account for longitudinal features that can be concatenated with an auxiliary input of all features such as demographics to be fed into a multilayer perceptron neural network with two or more hidden layers. During the modeling process, the data will be split into the observation window (e.g., 2005 to 2018) and the prediction window (e.g., 2018 to 2020) (Fig. 2). Data in the observation window will be used for model training, while those in the prediction window will be used for model evaluation. In the observation window, the dataset will be divided into training, validation, and testing sets with a ratio of 8:1:1, and the process will be iterated ten times. For each iteration, we will evaluate the model performance by means of the area under the receiver operator characteristic curve (AUC-ROC), and the 95% confidence interval will be used as an overall index of the diagnostic performance of our models.

For each model, we will then calculate mean precision (positive predictive value), sensitivity (recall, true positive rate), specificity (true negative rate), Youden’s index, AUC and Matthews Correlation Coefficient (MCC). The positive predictive value is defined as the proportion of participants who are correctly classified as fallers by the algorithm. Sensitivity is defined as the ratio of the number of fallers correctly classified to the total number of fallers and specificity is the ratio of the number of non-fallers correctly classified to the total number of non-fallers. Youden’s index and AUC can measure the effectiveness of a dichotomous diagnostic test and MCC score measures the quality of classification models. The optimal threshold of Youden’s index or AUC can be determined through sensitivity, specificity, and MCC.

#### Multitask learning architecture

**Fig. 3:**
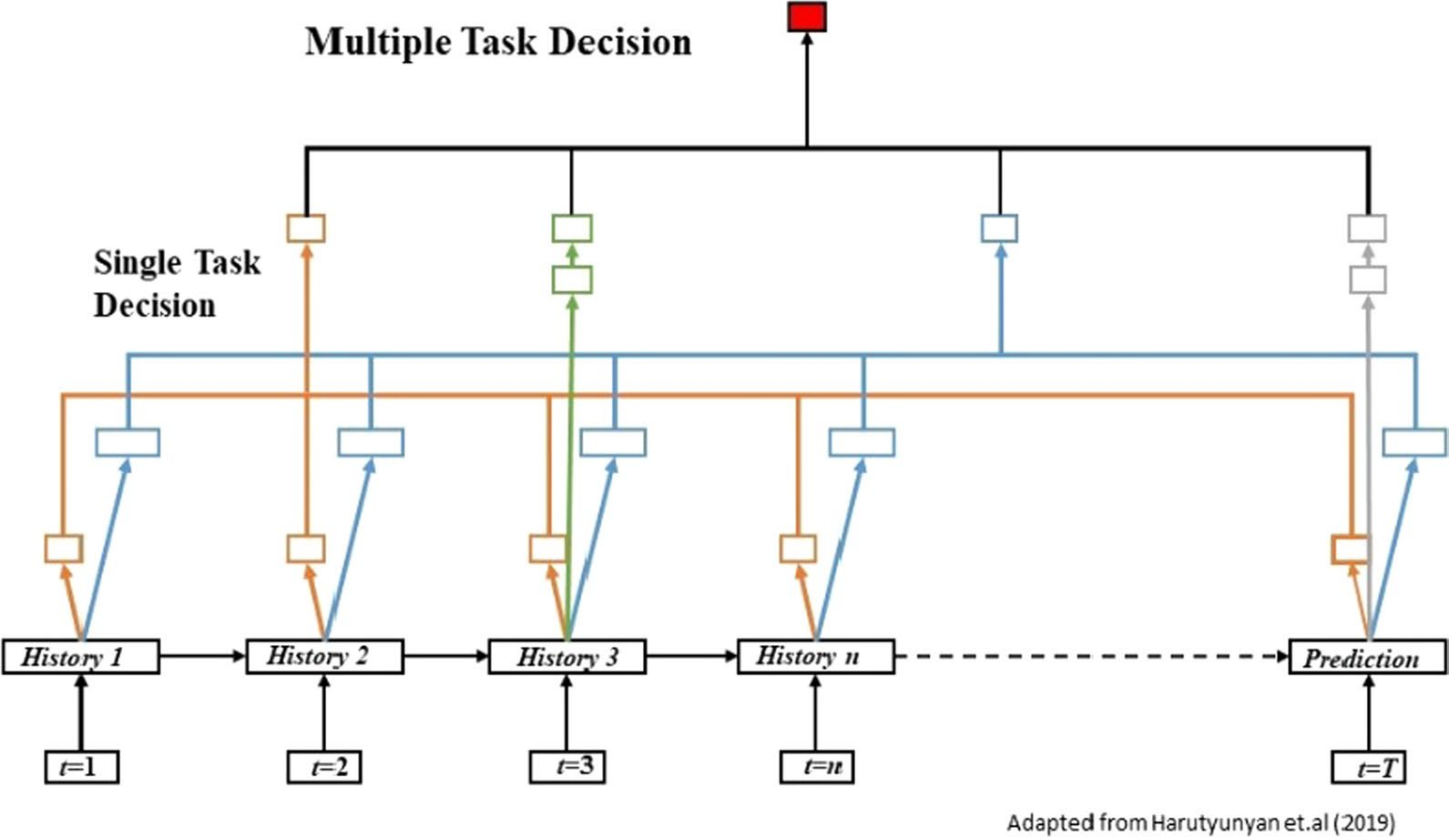
Multiple task learning architecture

Once we have the single prediction model, we will conduct the multitask learning architecture with LSTM modules (Fig. 3). A promising direction of multitask learning is to dynamically adapt these coefficients during training, similar to the adaptation of learning rates in optimizers. Multitask learning allows us to extract certain useful information from the input sequences that single-task models could not leverage and illustrates the better performance in some settings compared to a single task model.

### Phase 3: translational research

**Fig. 4:**
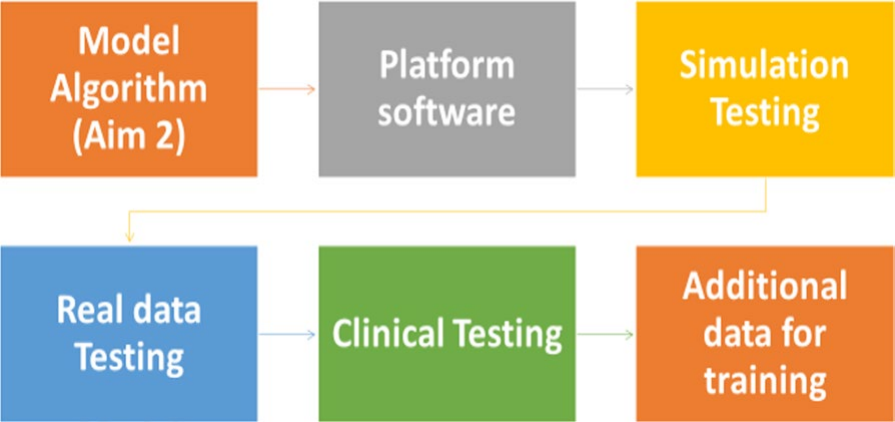
Process of translational phase

This phase targets the development of a prototype system to demonstrate the feasibility of implementing the developed risk prediction model in a clinical setting. Figure 4 depicts the process for implementing our AI-algorithm for VL level prediction and providing data driven evidence for clinical consultation. The testing system will be developed as a mobile application (app) that can be deployed on appropriate communication platforms for easy access in clinical environments by research assistants. We will use the Reactive Native cross-platform mobile app development framework for the prototype app building. The process is as follows: (1) We will first establish a database (2005–2020) in REDCap; (2) the prediction model will then be trained using the data; and (3) The system form will be established to link the data output from REDCap to our prediction models and generate the risk probability of viral predictor.

### Software development

**Fig. 5:**
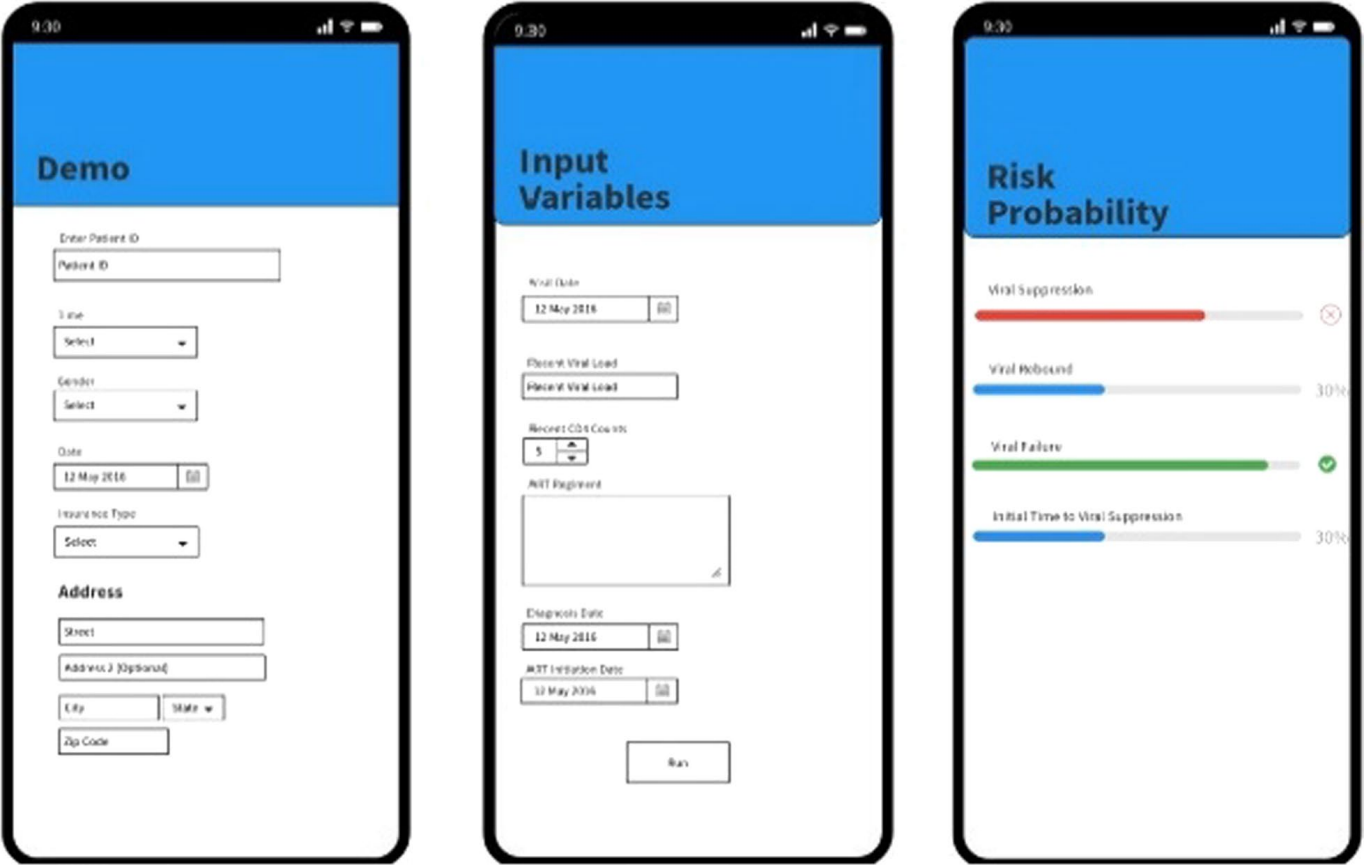
User interfaces of the prototype for viral load (VL) level prediction

In the software development stage, the trained medical chart reviewers will first extract the patient-level data from the EHR system (EPIC) and transfer the information to a REDCap database installed on a secure server and protected by the Secure Sockets Layer certification. Second, we will extract the data from REDCap to prediction platform. Generalizable middleware approaches will be employed for dynamic data pull of the integrated clinical and research data. The middleware approach can facilitate the adoption of REDCap dynamic data pull (DDP) module by institutions, and REDCap DDP has been widely used by investigators for integrating clinical and research data across the biomedical research enterprise. Third, we will develop the prototype system for VL prediction models. The clinicians and data specialists will collaborate with the interface developers to develop a user-friendly prototype system for the VL prediction model (see an example in Fig. 5). Lastly, we will develop a user manual with detailed instructions on installation and application of the developed software. The research team will work with clinicians to make the user manual easy to understand.

*Software Testing.* To test the feasibility of the software, we will first test the reliability of the platform in the simulation settings, where we will generate a simulated dataset to mimic the clinical setting to catch any error that might happen in practice. Second, we will test the accuracy of prediction using real clinical data. A research assistant will collect incoming patients’ data from the real clinic in EIPC software and then apply our system for prediction. The testing phase will last 3–4 months, and the research team will identify any issues during this testing phase and will meet and discuss how to improve this beta version. Third, we will perform platform evaluation in a real clinical setting. The software and the VL level prediction system will be adopted by one of our collaborator’s clinics. The onsite testing will be carried out from 6 to 12 months to test: (1) the feasibility of implementing the prediction system in real clinical settings; and (2) the prediction accuracy of the prediction models. Iterative improvements will be conducted when necessary, during program development.

## Discussion

The increasing availability of electronic data, including electronic medical records, administrative databases, and public county-level data, has created a unique opportunity to expand our ability to measure HIV-related health and clinical outcomes. With the integrated data, we can examine patients’ risk factors at the individual and county levels longitudinally and generate new knowledge of HIV viral suppression. However, these integrated data sources are characterized by high volume and variation, and there are several data analytic challenges in the integrated data structure, including mismatched time scales and multilevel risk predictors. The recent developments in Big Data analytics, such as artificial neural network [50, 51], LSTM Neural Network, random forest [51, 52], support vector machine [51], and deep learning approach such as CNN [53], make it feasible to address these methodological challenges and predict virologic outcomes using data from multiple domains.

The proposed project will integrate complex yet representative population-level HIV data from multiple SC data sources at both individual and county levels and analyze the impact of historic HIV VL patterns on multiple viral outcomes considering multilevel factors. The integrated data from these multiple data sources include PLWH who were diagnosed with HIV (as early as 2005) over a period of > 15 years (to at least 2020) and thus provide us a so-called synthetic cohort which contains a complete population-based longitudinal picture of HIV VL measure, HIV treatment, HIV care-seeking behaviors, hospital diagnosis, and county-level factors. We will develop AI-based modeling accounting for multiple viral outcomes by integrating both individual- and socioecological-level factors to relate it to future viral suppression. With both extensive data integration and Big Data analytics, the proposed research is significant as it will: (1) improve the understanding of the complex inter-related effects of longitudinal trajectories of HIV viral suppressions and HIV treatment history while taking into consideration multiple factors at the individual and socioecological levels among PLWH; and (2) develop empirical public health approaches to achieve ending the HIV epidemic through translating the risk prediction model to a multifactorial decision system that enables the feasibility of AI-assisted clinical decisions.

### Potential challenges

Overfitting can be a problem in deep neural networks with a large number of parameters. To avoid this problem, we will employ the dropout method [54], which is a common regularization technique for reducing overfitting in neural networks. The key idea is to randomly drop some neurons (along with their connections) from the neural network during training. This prevents the neural units from over co-adapting (note that dropout is disabled in testing, i.e., the whole network is used for estimation). For the integrated data we might have repeated observations at the single point or missing information at a particular measure. If there is more than one value available during a particular time point, the mean of the values during that time point will be calculated. If there is no value reported during a particular time, a missing value will be set. We will use mean and standard deviation to transform real values into categorical values; missing values will be assigned to a special category. Depending on the modelling approach, we will input the missing values.

### Ethics and dissemination

This study is approved by the University of South Carolina (UofSC) Institutional Review Board (Pro00109797**)**. The identity of all PLWH in the study is protected and only deidentified data will be released to the study researchers. The SC RFA will coordinate the efforts among relevant state agencies (e.g.SC DHEC, Health Sciences South Carolina [HSSC]) to link the data and to provide the study with only the deidentified data for analysis. Extensive data agreements ensuring data security and patient confidentiality for the deidentified linked data have been established and are stringently adhered to. No investigators will have access to identifiable data from any of the state agencies.

### Dissemination of results

To materialize the anticipated methodological and clinical benefits of the proposed research, and to maximize their impact on HIV clinical care, we will use the following strategies to disseminate the study findings: (1) Local Community and Stakeholder Forums. We will hold meetings with state agencies, including SC DHEC and RW to present study findings and prepare a data-driven strategic dissemination plan for local health care systems; and (2) Scientific Communities. Study dissemination will occur through presentations at academic conferences and the publication of peer-reviewed articles. We will capitalize on social media and professional networks that can increase the reach and accessibility of findings such as open access publication, webinars, files, and videos available on websites and publicly available channels to increase the visibility and impact of the scientific publications and presentations.

## Data Availability

Not applicable at this stage.

## Abbreviations

PLWH: People living with HIV
SC: South Carolina
EHR: Electronic Health Records
eHARS: Enhanced HIV/AIDS reporting system
RFA: The SC Office of Revenue and Fiscal Affairs
CDC: The Centers for Disease Control and Prevention
EtHE: Ending the HIV epidemic
VL: Viral load
VF: Virological failure
LLV: Low-level viremia
ART: Antiretroviral therapy
PI: Protease inhibitor
AI: Artificial intelligence
DHEC: Department of Health and Environmental Control
HSSC: Health Sciences South Carolina
SCDMH: SC Department of Mental Health
DAODAS: SC Department of Alcohol and Other Drug Abuse Services
ACS: American Community Survey
AHRF: Area Health Resources File
BRFSS: Behavioral Risk Factor Surveillance System
NRTI: Nucleoside/nucleotide reverse transcriptase inhibitors
NNRTI: Non-Nucleoside/nucleotide reverse transcriptase inhibitors
DDP: Dynamic data pull
CNN: Convolutional neural network
LSTM: Long short-term memory neural network
MCC: Matthews correlation coefficient
IRB: Institutional Review Board
HIPAA: Health_InsuranceHealth Insurance Portability and Accountability Act
